# Medium controlled redox activity and coordination behavior of a triazole indolinone ligand with vanadyl lons

**DOI:** 10.1038/s41598-026-44306-w

**Published:** 2026-04-02

**Authors:** Abdullah H. Mannaa, Esam A. Gomaa, Rania R. Zaky, Eslam A. Ghaith, Mahmoud N. Abd El-Hady

**Affiliations:** Chemistry Department, Faculty of Science, Mansoura University, Mansoura, Egypt

**Keywords:** Reversibility, Tafel plot, Complexation interaction, Stability constant, Job’s method, Molar ratio, Chemical biology, Chemistry

## Abstract

The electrochemical behavior of 3-(2-(4-amino-5-mercapto-*4 H*-1,2,4-triazol-3-yl)hydrazono)indolin-2-one (H_2_TIS) was investigated by cyclic voltammetry at a glassy carbon electrode at 303.15 K. The redox response of H_2_TIS was found to be strongly medium dependent. In acidic medium (0.1 M HNO_3_), the anodic process is attributed to a ligand centered oxidation involving proton-coupled electron transfer, while the cathodic response corresponds to reduction of the indolin-2-one carbonyl group. In neutral medium (0.1 M KCl), the carbonyl moiety displays a quasi-reversible redox couple. Conversely, in an alkaline medium (0.1 M NaOH), only a single irreversible anodic peak is observed, which is attributed to the oxidation of the deprotonated thiolate moiety, plausibly leading to the formation of disulfide linked dimeric species. The electrochemical behavior of vanadyl ions (VO^2+^) was also studied in 0.1 M KCl, both in the absence and presence of H_2_TIS. The VO^2+^ system exhibits a quasi-reversible redox reaction under near-neutral conditions, while coordination with H_2_TIS induces potential shifts and increases the electrochemical stability of the metal center. Stoichiometric analysis based on cyclic voltammetry, Job’s method and molar ratio plots suggest formation of 1:1 and 1:2 VO^2+^-H_2_TIS complexes in solution. These findings highlight the medium-dependent redox versatility of H_2_TIS and the promising electrochemical properties of the VO^2+^ complexes.

## Introduction

The interactions between organic ligands and metal ions play an important role in coordination chemistry, due to their wide applications in catalysis, sensing, environmental monitoring and medicinal chemistry^1,2^. Among these ligands, thiazole- and triazole-based chelating agents, such as H_2_TIS (3-(2-(4-amino-5-mercapto-4 H-1,2,4-triazol-3-yl)hydrazono)indolin-2-one) have recently attracted considerable attention for their ability to form stable coordination complexes with transition-metal ions *via* sulfur, nitrogen and oxygen donor sites^3,4^. Recent studies on metal(II)-triazole complexes have shown different coordination modes with nitrogen and sulfur donor atoms as well as distinct electronic properties, as revealed by spectroscopic analysis and DFT calculations^5,6^.

Electrochemical characterization using cyclic voltammetry (CV) is a powerful approach to investigate redox active ligands and their metal complexes^7^. Through this technique, valuable information can be obtained regarding electron transfer kinetics, reversibility and stability of ligand-metal systems under different conditions. In this study, the redox properties of H_2_TIS ligand are investigated in the presence of vanadyl ions (VO^2+^) in media a 0.1 M HNO_3_, NaOH and KCl. Redox processes, including oxidation of the amino group to the nitro group and reduction of the carbonyl group in acidic and neutral environments, provide mechanistic insight into the electrochemical reactivity and stability of the system^8^.

In parallel with the electrochemical studies, spectrophotometric investigations were performed to determine the stoichiometry and stability of the resulting complexes. Both Job’s continuous variation and the molar ratio methods were employed to identify the predominant 1:1 and 1:2 metal-ligand stoichiometries^9–11^. The spectrophotometric results complement the CV data, confirming complex formation through changes in absorbance as a function of concentration. Furthermore, the calculated stability constants (β) and Gibbs free energy values (ΔG) revealed the spontaneous nature of the coordination process, underlining the thermodynamic stability of the VO^2+^-H_2_TIS complexes^12^. Such combined electrochemical spectrophotometric approaches are increasingly recognized as robust tools for clarifying coordination mechanisms and predicting catalytic or sensing behavior^13^. The motivation for the present research lies in the urgent demand for efficient coordination compounds capable of selective metal-ion detection and catalytic activity, particularly in environmental and biological systems^14,15^. Vanadium compounds, in particular exhibit rich redox chemistry and biological relevance, and their interaction with triazole and thiosemicarbazone based ligands offers promising routes for designing redox active materials and electrochemical sensors^16,17^. Vanadyl ions (VO^2+^) are particularly suitable for coordination with multidentate triazole based ligands due to their well-defined square pyramidal geometry, strong oxophilicity and accessible V(IV)/V(V) redox couple. The presence of mixed sulfur, oxygen and nitrogen donor sites in the H_2_TIS ligand is expected to promote selective and stable coordination with VO^2+^, leading to pronounced electrochemical responses and enhanced complex stability. Previous studies on vanadylazole systems have highlighted their potential in redox sensing and catalytic applications; However, comprehensive electrochemical spectrophotometric investigation combined with theoretical analysis is limited^18^. The present work addresses this gap by elucidating the coordination and redox behavior of the VO^2+^-H_2_TIS system, leading to its potential application in metal ion sensing and catalysis. Understanding the interactions between H_2_TIS and VO^2+^ ions thus contributes to developing new multifunctional complexes with potential applications in catalysis, metal ion sensing and environmental remediation^19,20^.

By integrating cyclic voltammetry, spectrophotometric analysis, and theoretical calculations, this study provides a comprehensive understanding of the coordination and electronic behavior of the H_2_TIS and its vanadyl complex. The insights obtained here advance the design of next generation ligand based electrochemical materials and sensors for practical industrial and environmental applications^21^.

## Experimental

### Cyclic voltammetry procedure

In this work, sodium hydroxide (NaOH), nitric acid (HNO_3_), potassium chloride (KCl), vanadyl sulfate pentahydrate (VOSO_4_.5H_2_O) and dimethyl sulfoxide (DMSO) were purchased from Merck and Sigma Aldrich and used without further purification.

Cyclic voltammetry measurements were conducted using a DY 2100 potentiostat in a conventional three electrode system, consisting of a glassy carbon working electrode (GCE), an Ag/AgCl reference electrode and a platinum wire counter electrode. The electrochemical behavior of the H_2_TIS was investigated in 0.1 M aqueous solutions of NaOH, HNO_3_ and KCl. Additionally, the redox behavior of the VO^2+^-H_2_TIS complex was studied in 0.1 M KCl. Cyclic voltammetry experiments were conducted at a scan rate of 0.05 V.s^− 1^ to investigate concentration effects. For scan rate studies, measurements were additionally performed at 0.01, 0.02, 0.05 and 0.10 V.s^− 1^ using the final ligand concentration. All experiments were carried out at 303.15 K following IUPAC conventions for electrode potential reporting^22–24^.

### Spectrophotometric analysis

#### Job’s method (continuous variation)

Job’s method of continuous variation was employed to study the interaction between VO^2+^ ions and the H_2_TIS ligand. A series of solutions was prepared by mixing equimolar concentrations of the metal and ligand in varying molar ratios, while keeping the total concentration constant at 1 × 10^− 3^ M. All experiments were conducted at room temperature (303.15 K). The absorbance of each solution was measured at the wavelength corresponding to maximum absorbance (λ max), and a plot of absorbance versus the mole fraction of the metal ion exhibited a maximum, indicating the most stable metal to ligand stoichiometric ratio in solution^25,26^.

#### Molar ratio method

In this method, the concentration of VO^2+^ ions was kept constant, while the concentration of the H_2_TIS ligand was varied systematically. The absorbance of each solution was measured at the maximum wavelength (λ max = 490 nm) at room temperature (303.15 K). A plot of absorbance versus the ligand to metal molar ratio was constructed, and the point of intersection of the extrapolated linear segments indicated the most stable stoichiometric ratio of the VO^2+^ complexes formed in solution^27^.

## Results and discussions

### Cyclic voltammetry studies

#### Electrochemical behavior of H_2_TIS ligand in 0.1 M HNO_3_ medium

**Scheme 1:**
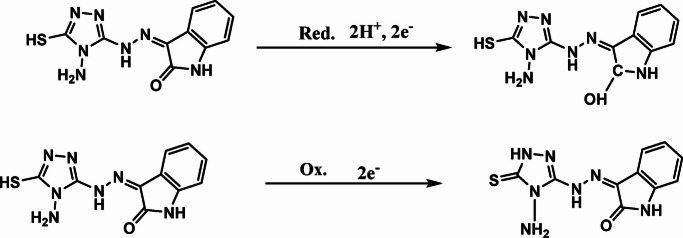
Proposed electrochemical reduction and oxidation mechanisms of the H_2_TIS ligand in 0.1 M HNO_3_.

**Fig. 1:**
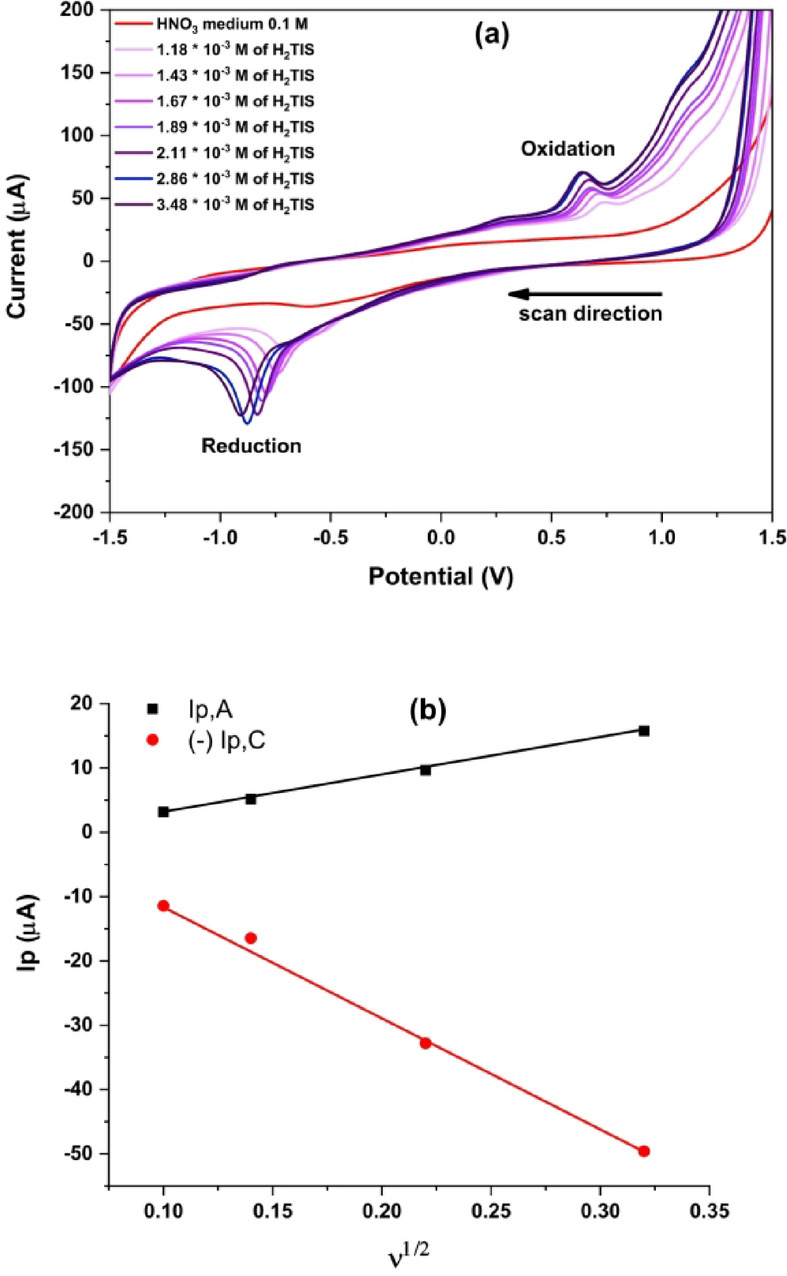
**(a)** Cyclic voltammograms of different H_2_TIS ligand concentrations in 0.1 M HNO_3_ recorded at 0.05 V.s^− 1^ and (**b**) Plot of peak current (Ip) *versus* the square root of scan rate (ʋ^1/2^) obtained at scan rates of 0.01–0.10 V.s^− 1^ using the final ligand concentration.

The cyclic voltammetry of the H_2_TIS ligand 3.48 × 10^− 3^ mol L^− 1^ in 0.1 M HNO_3_ (pH 1.5) was recorded at a glassy carbon electrode within a potential window of + 1.5 to −1.5 V at a scan rate of 0.05 V.s^− 1^ (303.15 K), as shown in Fig. 1a. A cathodic peak was observed in the potential range of −0.68 to −0.91 V and is tentatively assigned to a ligand centered reduction process involving the indolin-2-one carbonyl group (C = O). The slight of the cathodic peak toward more negative potentials with increasing ligand concentration, together with the absence of a well-defined reverse peak, supports an irreversible to quasi-irreversible reduction pathway under these acidic conditions. In line with Scheme 1, this cathodic response is consistent with a proton coupled, two electron reduction of the lactam carbonyl (C = O→C-OH), yielding a reduced carbinolamide (hemiaminal) product^28^.

An anodic peak was observed within the potential range of 0.64–0.74 V. In acidic medium, the anodic response is more plausibly attributed to oxidation at the sulfur containing site of the ligand. As illustrated in Scheme 1, the anodic process is proposed to involve oxidation of the thiol functionality (-SH), leading to thiyl type intermediates, which rationalizes the predominantly irreversible anodic behavior^29^.

Notably, the peak currents increased with increasing ligand concentration. Moreover, the linear dependence of both anodic and cathodic peak currents on the square root of the scan rate (Ip α ʋ^1/2^; Fig. 1b) indicates that mass transport is predominantly diffusion controlled at the planar electrode surface under the applied conditions. To evaluate the mass transport mechanism, cyclic voltammetry was performed at scan rates of 0.01, 0.02, 0.05 and 0.10 V.s^− 1^ at the final ligand concentration. The linear dependence of peak current on the square root of scan rate (Ip ∝ ʋ^1/2^), in accordance with the Randles-Sevcik equation^30^, confirms diffusion-controlled behavior. Overall, the combined features of peak shifts, lack of a fully developed reverse wave, and the proposed chemical follow-up steps are consistent with an EC type mechanism, as summarized in Scheme 1.

#### Job’s method electrochemical behavior of H_2_TIS ligand in 0.1 M NaOH medium

**Fig. 2:**
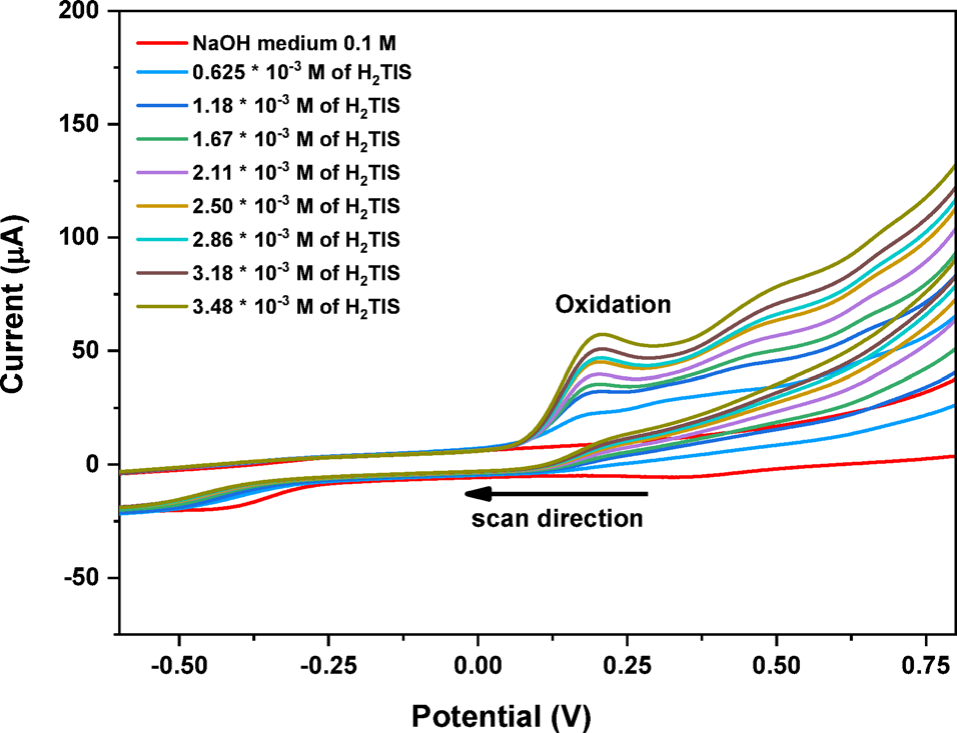
Cyclic voltammograms of different (H_2_TIS) ligand concentrations in 0.1 M NaOH.

The cyclic voltammetry of the H_2_TIS ligand (3.48 × 10^− 3^ mol.L^− 1^) in 0.1 M NaOH (pH = 12.5) was recorded within a potential window of + 0.75 to −0.50 V at a scan rate of 0.05 V.s^− 1^ (Fig. 2). A single anodic peak was observed in the potential range of 0.18–0.21 V, with the peak current increasing as a function of ligand concentration. The anodic peak potential remains essentially constant upon increasing ligand concentration, and the absence of a corresponding cathodic peak confirms the irreversible nature of the oxidation process under alkaline conditions.

**Scheme 2:**
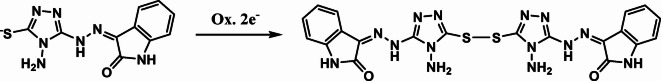
Proposed oxidation pathway of the H_2_TIS ligand in 0.1 M NaOH.

Under strongly alkaline medium, the thiol group of the H_2_TIS ligand is expected to be predominantly deprotonated to form a thiolate species (L-S^−^), which is more readily oxidized than the protonated thiol. Accordingly, the observed anodic response is more plausibly attributed to electrochemical oxidation of the thiolate moiety, followed by coupling of sulfur centered intermediates to yield disulfide linked dimeric species^31–33^, as schematically illustrated in Scheme 2.

#### Electrochemical behavior of H_2_TIS ligand in 0.1 M KCl medium

**Scheme 3:**
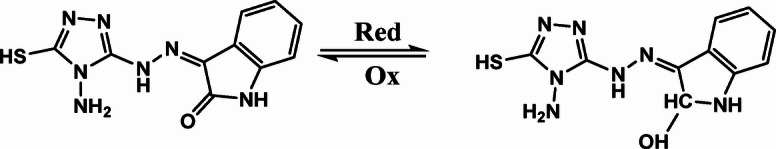
Suggestion Redox mechanism of H_2_TIS ligand in 0.1 M KCl.

**Fig. 3:**
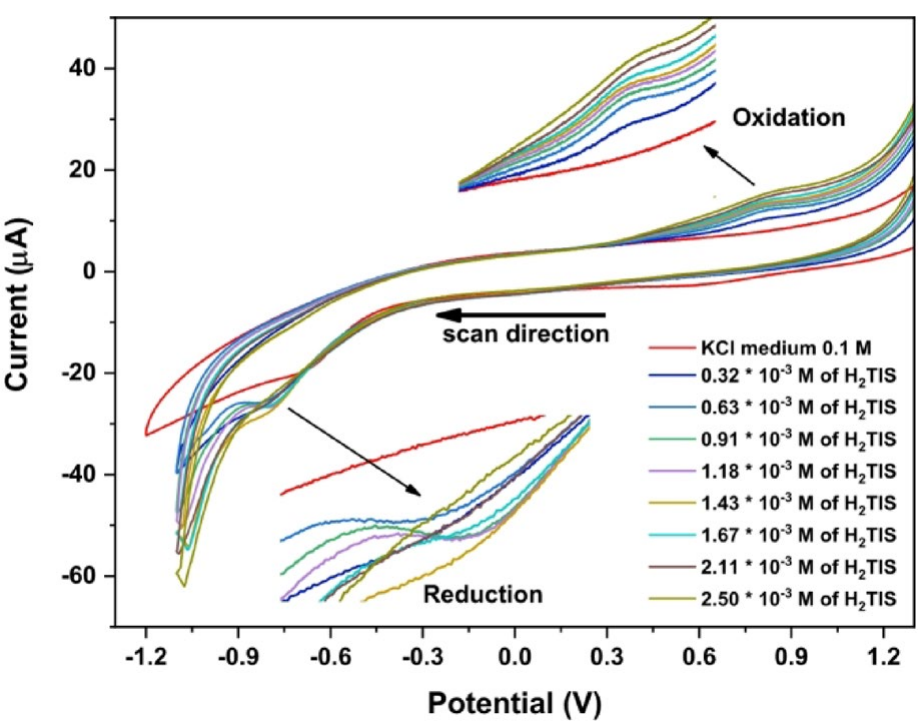
Cyclic voltammograms at varying H_2_TIS concentrations in 0.1 M KCl.

The redox reactions of the H_2_TIS ligand at a concentration of 2.5 × 10^− 3^ mol.L^− 1^ in 0.1 M KCl (pH = 6.8) was conducted under stable conditions, using a potential window of + 1.2 to −1.2 V, a scan rate of 0.05 V.s^− 1^, and at room temperature (303.15 K), displayed in Fig. 3. Two peaks in a quasi-reversible reduction and oxidation process of the carbonyl group were observed in a potential range of −0.78 to −0.84 V, and + 0.80 to + 0.85 V, respectively. The reduction and oxidation peak potentials show only minor variation with changing experimental conditions, while the moderate peak separation indicates a quasi-reversible redox process for the carbonyl group in neutral medium. The proposed mechanism for the H_2_TIS ligand’s oxidation behavior in neutral medium is shown in Scheme 3.

#### Comparative Electrochemical Behavior of H_2_TIS in Different Media

The redox response of H_2_TIS varies markedly with the surrounding medium. In acidic solution (HNO_3_), both reduction and oxidation peaks appear due to proton-assisted electron transfer, while in neutral KCl, a quasi-reversible couple indicates a balanced proton-coupled mechanism. Under basic NaOH, only a single irreversible oxidation peak is detected, reflecting ligand centered oxidation of the deprotonated species. This medium-dependent behavior demonstrates that proton availability controls the electron transfer pathways of H_2_TIS ligand, and underscores the potential for pH-sensitive sensors and redox-based catalytic systems.

#### Electrochemical behavior of VO^2+^-H_2_TIS system

To explore the interaction between H_2_TIS and vanadyl ions (VO^2+^), CV measurements were performed in solutions containing different concentrations of VO^2+^, and the resultant voltammograms were analyzed for changes in peak position and intensity. In general, the ligand H_2_TIS is often characterized by specific functional groups that can coordinate with metal centers.

#### Theoretical calculations

The diffusion coefficient of the reduced (D_a_) and oxidized (D_c_) species was intended from the Randles-Ševčík Eq. (1)^30^

1 $${{\mathrm{i}}_{\mathrm{p}}}={\text{ }}(0.4463{\text{ }}{{\mathrm{n}}^{3/2}}{{\mathrm{F}}^{3/2}}{\mathrm{AC}}{{\mathrm{D}}^{1/2}}{{{\upnu}}^{1/2}}){\text{ }}/{\text{ }}{\left( {{\mathrm{RT}}} \right)^{1/2}}$$

Where ip is the current, n is the number of electrons transferred in the redox reaction, F is the Faraday constant (96485.33 coulombs), A is the GCE surface area (0.0314 cm^2^), ν is the scan rate (mV.s^− 1^), and C is the VO^2+^ ion concentration.

The heterogeneous electron transfer rate constant (k_h_) was determined using the Klinger-Kochi Eq. (2)^34,35^

2 $${{\mathrm{k}}_h}={\text{ }}2.18^*{\left[ {{\text{F }}\alpha {\text{ }}{{\mathrm{n}}_{\mathrm{a}}}{{\mathrm{D}}_{\mathrm{C}}}\upnu /{\mathrm{RT}}} \right]^{1/2}}\:^*{\text{exp }}\left[ {{\text{F }}{\alpha ^2}{\text{n }}\Delta {{\mathrm{E}}_{\mathrm{P}}}/{\mathrm{RT}}} \right]$$

Where: α and n_a_ are the coefficients of charge transfer and the electron transfer number in the rate determining step, respectively.

The number of electrons participating in the rate-determining step (n_a_) can be determined *via* Eq. (3)^7^

3 $${{\mathrm{n}}_{\mathrm{a}}}=1.857{\text{ RT }}/{\text{ }}\left( {{{\mathrm{E}}_{{\mathrm{pc}}}} - {{\mathrm{E}}_{{\mathrm{pc}}/2}}} \right){\text{ }}\alpha {\text{ F}}$$

Surface coverage concentration (charge quantity) on the working electrode was calculated using Eq. (4)^36^

4 $$\Gamma {\text{ }}={\text{ }}{{\mathrm{i}}_{\mathrm{p}}}4{\text{RT }}/{{\mathrm{n}}^2}{{\mathrm{F}}^2}{\text{A }}\upnu$$

The quantities of charge used during oxidation and reduction at the working electrode surface were evaluated using Eq. (5)^37^

5 $${\text{Q }}={\text{ n FA }}\Gamma$$

#### Electrochemical properties of free VO^2+^ ions

**Fig. 4:**
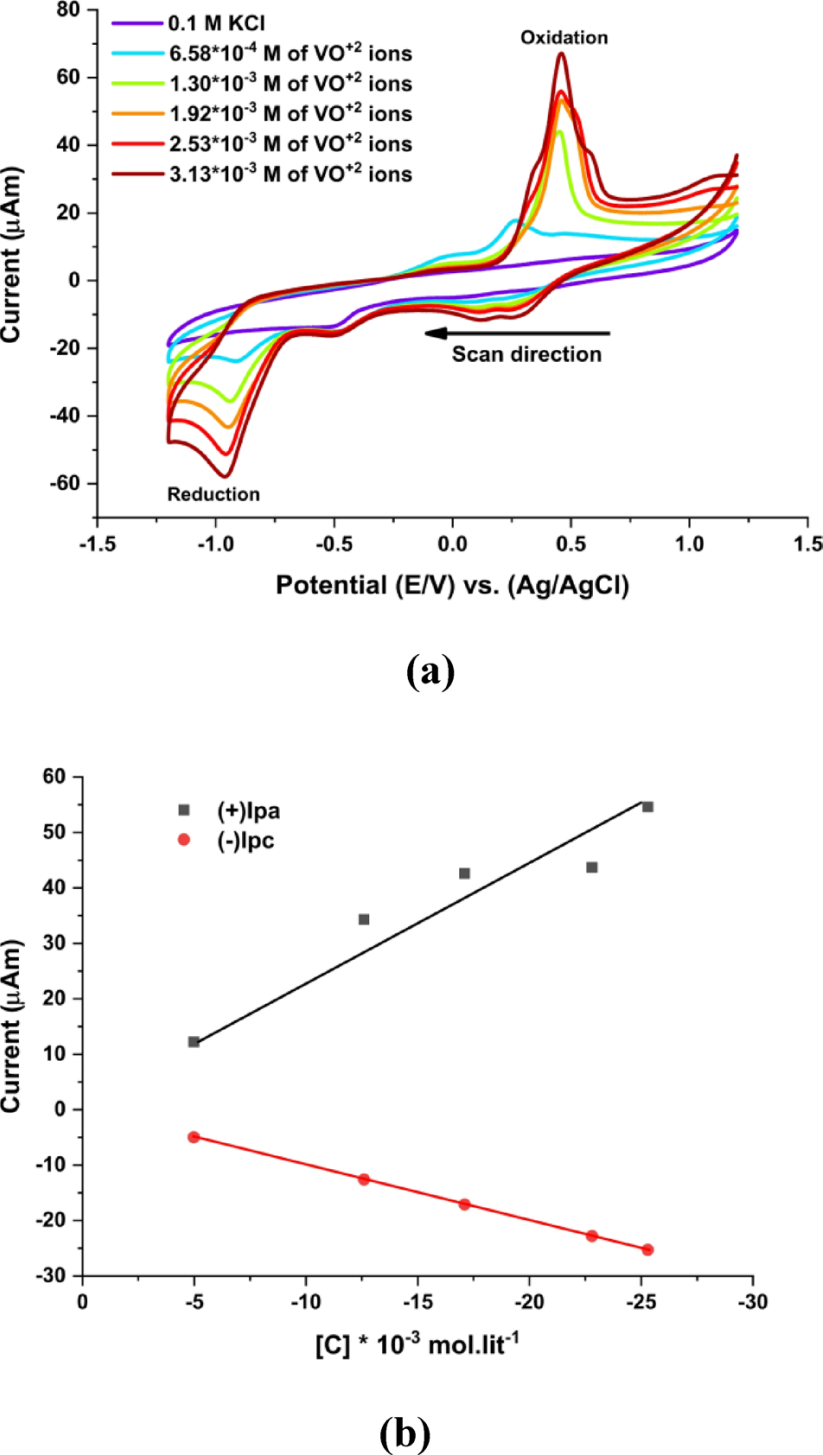
**(a)** Cyclic voltammograms of VO^2+^ at increasing molar concentrations (6.58 × 10^− 4^ to 3.13 × 10^− 3^ M) in 0.1 M KCl at 303.15 K. (**b)** Relationship between cathodic anodic and peak heights for increasing VO^2+^ ions concentrations.

The electrochemical properties of a 3.13 × 10^− 3^ M VO^2+^ cation were investigated by cyclic voltammetry using a glassy carbon working electrode (GCWE) in a 0.1 M KCl supporting electrolyte under aqueous conditions. The measurements were carried out within a potential window from + 1.2 to −1.2 V at a scan rate of 0.05 V.s^− 1^ and a temperature of 303.15 K, as shown in Fig. 4a. The voltammogram exhibited a cathodic peak during the forward scan and a corresponding anodic peak during the reverse scan, which can be attributed to a VO^2+^ centered redox process involving the electrochemical reduction of VO^2+^ to a lower oxidation state. The stability and structural features of VO^2+^ in aqueous solution have been well documented in the literature^38^. The anodic and cathodic peak potentials exhibit limited variation with concentration, with Epa ranging from 0.27 to 0.46 V and Epc from − 0.91 to −0.96 V (Table 1), while the large peak separation and non-unity ipa/ipc ratio support a kinetically controlled quasi-reversible VO^2+^/V^2+^ redox process in near-neutral aqueous medium. The associated redox reaction can be represented by Eq. (8).

6 $$\mathrm{VO}^{2+} + 2\mathrm{H}^+ + 2\mathrm{e}^- \rightleftharpoons \mathrm{V}^{2+} + \mathrm{H}_2\mathrm{O}$$

Importantly, under-near neutral aqueous conditions, the reduced vanadium species generated electrochemically is expected to be transient and may undergo rapid follow up chemical processes such as oxidation by dissolved oxygen or hydrolysis. This behavior is consistent with previous reports describing the high reactivity and limited stability of V^2+^ species in aqueous media^39^. Such limited stability is reflected in the significant deviation of the apparent peak-to-peak separation (ΔEp) and anodic to cathodic peak current ratio (IPA/IPC) from unity, indicating a clear quasi-reversible electrochemical behavior with noticeable kinetic limitations under the experimental conditions used. Cyclic voltammetric parameters including cathodic and anodic peak potentials (Epa and Epc) and peak currents (Ipa and Ipc) were used to evaluate the reversibility and kinetic properties of the system. These parameters enabled the calculation of ΔEp, current ratio (Ipa/Ipc), number of transferred electrons (Na) and heterogeneous electron transfer rate constant (Kh), as summarized in Table 1. Furthermore, the anodic and cathodic charge amounts and surface coverage values (Table 2) reveal a clear oxidation and reduction process between reduction and reduction processes. the nature of the redox process. Furthermore, peak current was found to increase with increasing VO^2+^ concentration, as shown in Fig. 4b^40^.

#### Tafel plot analysis

**Fig. 5:**
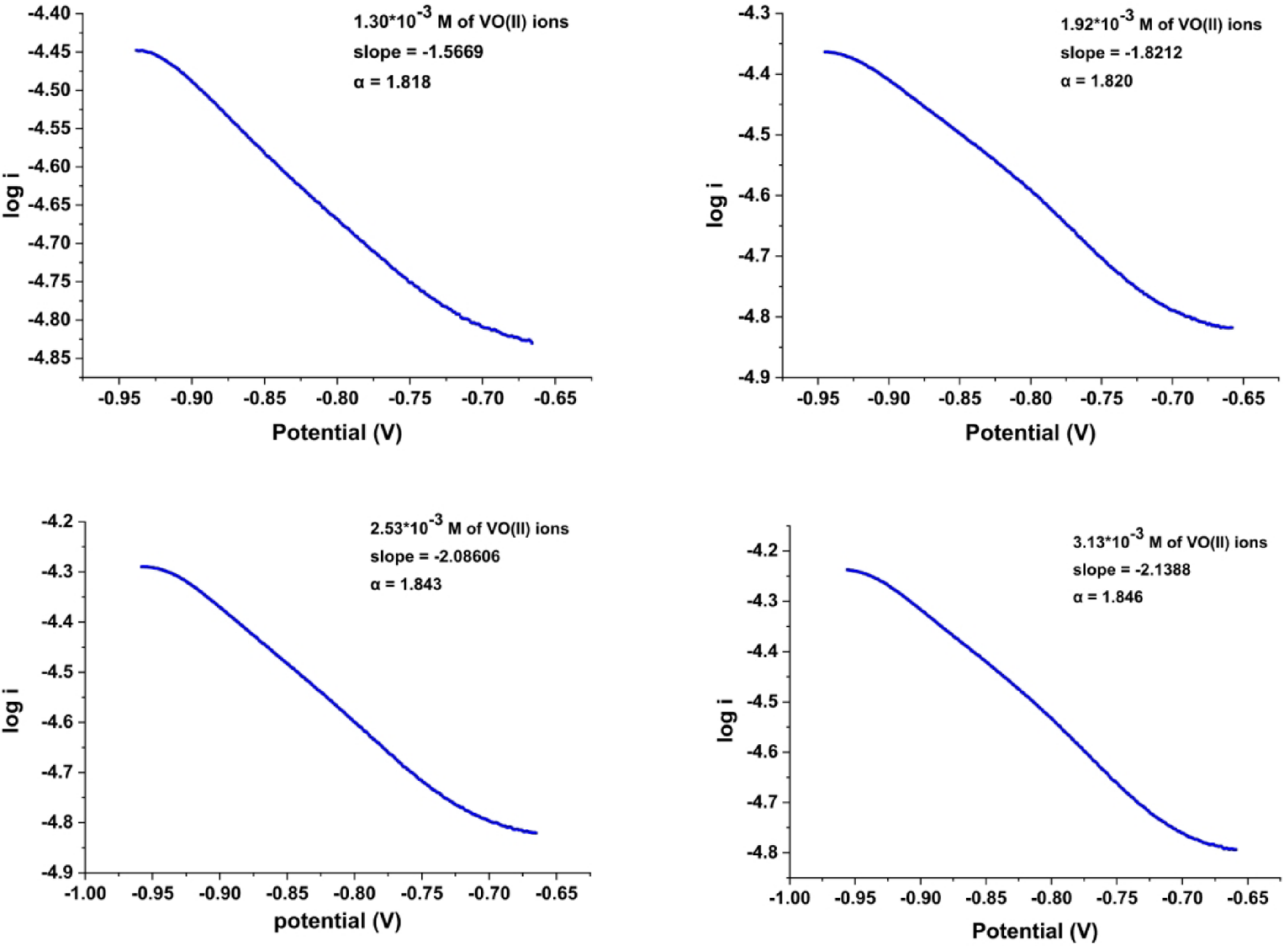
Tafel plot at different VO^2+^ ions concentrations (1.30 × 10^− 3^ to 3.13 × 10^− 3^ M).

The charge transfer coefficient (α) quantifies the reduction in the free energy barrier associated with electrochemical processes occurring at electrode-electrolyte interfaces. α was estimated using Tafel plot analysis (Fig. 5), a technique involving plotting log i versus potential. The slope of this plot, specifically the initial segment of the cathodic peak from cyclic voltammetry was used in Eq. (9) to calculate α^40^.

7 $$\mathrm{Slope} = -\alpha \text{F / RT}$$

The Tafel plots (Fig. 5) demonstrate that the slope values for all additions range from − 1.5669 to −2.1388, corresponding to charge transfer coefficients between 1.818 and 1.846, suggesting that the redox process for free VO^2+^ ions is irreversible.

All cyclic voltammetry parameters such as E_pc_, E_p.a._, ∆E_p_, i_pc_, i_p.a._, i_p.a._/i_pc_, D_c_, D_a_, E_pc/2_, E_pc_-E_pc/2_, k_h_, α, and n_a_ for the free ions of VO^2+^ is summarized in Table 1.

**Table 1:** Cyclic voltammetry data for VO^2+^ ions at 303.15 K.

[M] x10^− 3^ mol.L^− 1^	E_*p*.a._	E_pc_	∆E_*p*_	i_*p*.a._	-i_pc_	i_*p*.a._/i_pc_	D_a_	D_c_	E_pc/2_	E_pc_-E_pc/2_	Α	*n*_a_	k_h_ cm.sec^− 1^
	V			µA			cm^2^/sec x10^− 6^		V				
0.66	0.269	−0.911	1.18	12.2	4.98	2.440	12.40	2.083	−0.854	1.765	0.059	0.472	0.001
1.30	0.453	−0.941	1.394	34.3	12.6	2.727	25.34	3.408	−0.877	1.818	0.096	0.283	0.002
1.92	0.461	−0.946	1.407	42.6	17.1	2.491	17.84	2.874	−0.874	1.820	0.111	0.243	0.003
2.53	0.457	−0.956	1.413	43.7	22.8	1.918	10.84	2.947	−0.887	1.843	0.128	0.210	0.005
3.13	0.457	−0.958	1.415	54.6	35.3	2.157	11.08	2.381	−0.888	1.846	0.131	0.204	0.005

The values of Q and Γ for both oxidation and reduction reactions were evaluated for all VO^2+^ additions, as shown in Table 2. As the concentration of VO^2+^ ions in the cell rises, the surface coverage at the glassy carbon electrode increases, resulting in enhanced charge transfer between the solution and the GCE surface^41^.

**Table 2:** Anodic and cathodic surface coverage concentrations, along with charge quantities, for various VO^2+^ ion concentrations.

[M] x10^− 3^ mol/lit	I_*p*.a._ (µA)	-I_pc_ (µA)	Γ_c_ x10^− 9^ (mol. cm^− 2^)	(-) Q_c_ x10^− 5^ (columb)	Γ_a_ x10^− 9^ (mol. cm^− 2^)	(+) Q_a_ x10^− 5^ (columb)
0.658	12.2	4.98	0.873	0.529	2.131	1.29
1.30	34.3	12.6	2.205	1.34	6.014	3.64
1.92	42.6	17.1	2.999	1.82	7.472	4.53
2.53	43.7	22.8	3.998	2.42	7.666	4.64
3.13	54.6	35.3	4.436	2.69	9.568	5.80

#### Scan rate effects on the electrochemical properties of free VO^2+^ ions

**Fig. 6:**
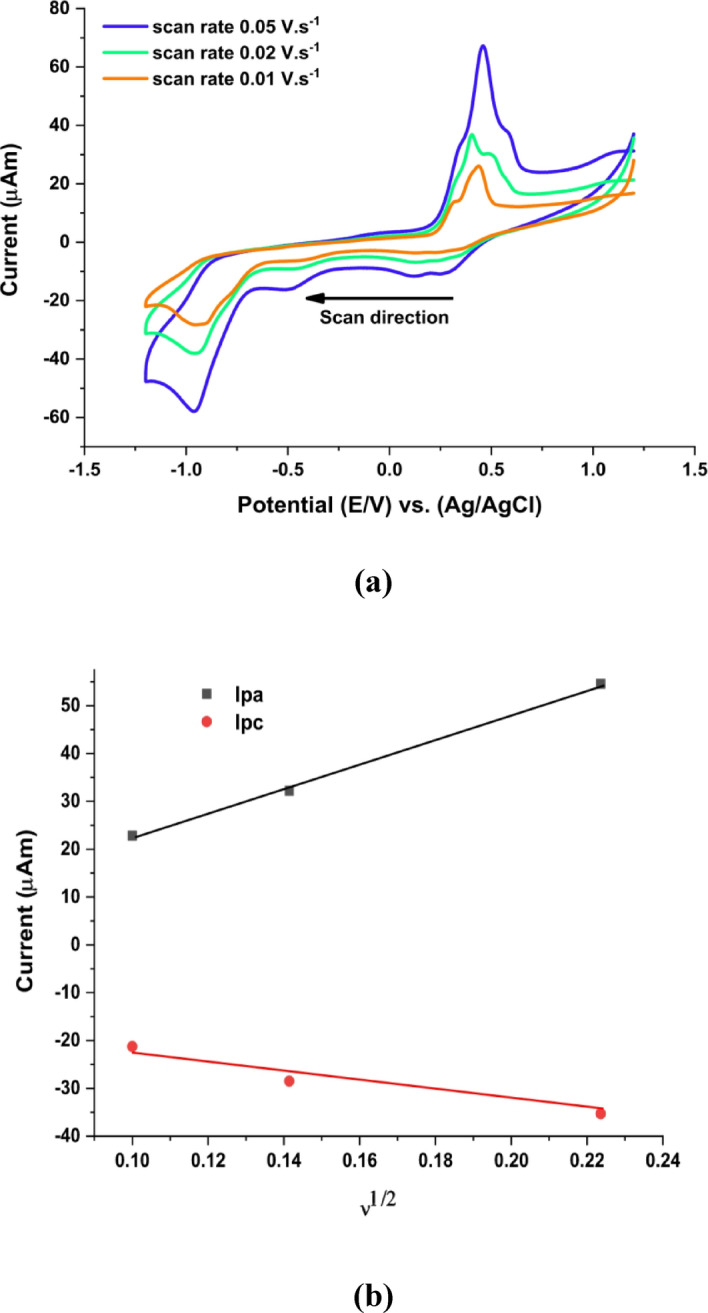
**(a)** Redox behavior of 3.13 × 10^− 3^ mol.L^− 1^ VO^2+^ ions at varying scan rates. **(b)** Ipa & Ipc vs. scan rate square root at 3.13 × 10^− 3^ mol.L^− 1^ of VO^2+^ ions.

Cyclic voltammetry was conducted for a 3.13 × 10^− 3^ mol.L^− 1^ solution of VO²⁺ at scan rates of 0.01, 0.02, and 0.05 V.s^− 1^ (Fig. 6a). The corresponding diffusion related parameters were estimated from the linear dependence of the anodic and cathodic peak currents (Ipa and Ipc) on the square root of the scan rate (ν^1/2^), as shown in Fig. 6b and summarized in Table 3. Although the classical Randles-Ševčík equation is strictly valid for reversible diffusion-controlled systems, its application here is used in a semi-quantitative manner to assess the predominance of diffusion in the mass transport process. The non zero intercepts observed in Fig. 6b indicate an additional contribution from non-faradaic (capacitive) currents and/or weak adsorption phenomena at the electrode surface, consistent with deviations from ideal reversibility.

As the scan rate increases, the anodic to cathodic peak separation (ΔEp) also increases, confirming the irreversible nature of the redox process. The charge-transfer coefficient (α = 0.13) and heterogeneous electron-transfer rate constant (kh = 0.012–0.025 cm.s^− 1^) fall within the range typically associated with kinetically hindered, irreversible electron-transfer systems. Furthermore, the increase in ΔEp (1.386 → 1.415 V) and the rise in the Ipa/Ipc ratio (1.07 → 2.16) with scan rate reflect increasing deviation from ideal reversibility and the predominance of the oxidation process at higher scan rates.

Despite the irreversible character of the redox process, the diffusion coefficients obtained for both anodic and cathodic processes remain on the order of 10^− 6^ cm^2^.s^− 1^, indicating that mass transport is largely governed by diffusion. Therefore, the electrochemical behavior of VO^2+^ species can be described as diffusion-controlled with significant kinetic limitations, highlighting the interplay between charge transfer kinetics and mass transport processes in defining the overall electrochemical response.

**Table 3:** Cyclic voltammetry data were obtained for a 3.13 × 10^− 3^ mol.L^− 1^ of VO^2+^ ions at different scan rates.

Scan rate (V.s^− 1^)	E_*p*.a._	E_pc_	∆E_*p*_	i_*p*.a._	(-)i_pc_	i_*p*.a._/i_pc_	D_a_	D_c_	E_pc/2_	E_pc_-E_pc/2_	Α	*n*_a_	k_h_ cm.sec^− 1^
	V			µA			cm^2^/sec x10^− 6^		V				
0.05	0.457	−0.958	1.415	54.6	35.3	2.157	11.08	2.381	−0.888	1.846	0.131	0.204	0.025
0.02	0.404	−0.947	1.351	32.2	28.5	1.130	9.635	7.551	−0.824	1.771	0.129	0.215	0.014
0.01	0.437	−0.949	1.386	22.8	21.3	1.073	9.694	8.415	−0.784	1.733	0.133	0.213	0.012

**Fig. 7:**
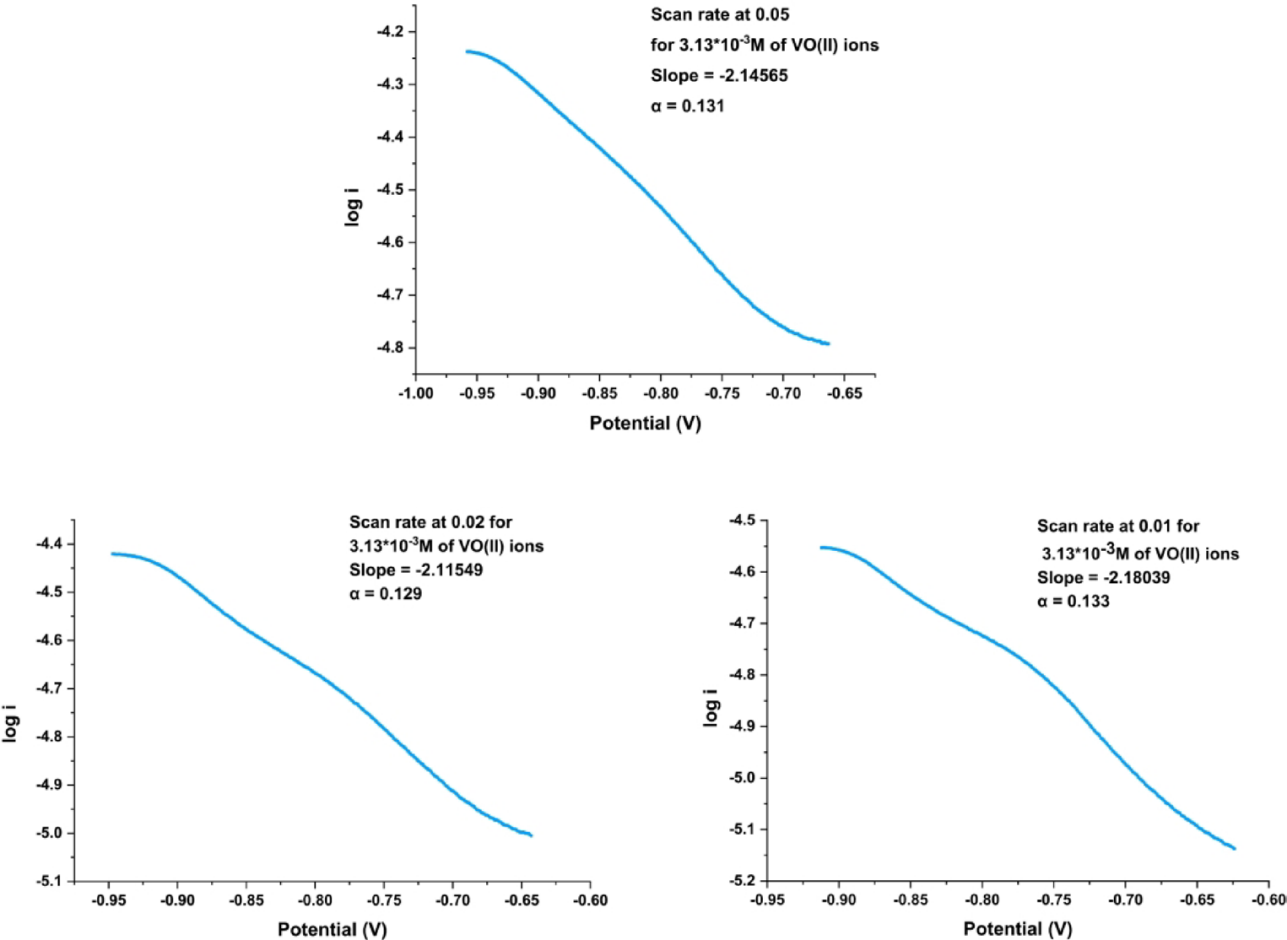
Effect of scan rate difference (0.05, 0.02 and 0.01 V.s^− 1^) on the Tafel plot following the final VO^2+^ ion addition.

Tafel plots (Fig. 7) illustrate that the slopes across changed scan rates range from − 2.115 to −2.180 which corresponds to charge transfer coefficients between 0.129 and 0.133. This shows an irreversible electrochemical process (Table 3). The Tafel plot slopes can change with scan rate; at increased scan rates, the Tafel slope may appear steeper, suggesting that activation processes predominantly change the reaction kinetics over mass transport. At lower scan rates, the slopes tend to be shallower, representing that diffusion limitations have a more pronounced effect on the reaction kinetics.

Examining variations in Tafel slopes and current densities at different scan rates provides insight into fundamental reaction processes. If the slopes reflect a single-electron transfer pathway, this means that the reaction mechanism is the same at different scan rates, even though they are affected by the presence of VO^2+^. Analysis of the slopes and current densities allows inferences about the properties of the electrochemical reaction and the influence of VO^2+^ on its kinetics.

#### Influence of scan rate on adsorption dynamics and charge quantity

The effects of scan rate on charge quantities and adsorption were investigated. On the GCE surface, the surface coverage increased as the scan rate decreased, which was due to the longer duration of the redox process. This indicates that the amount of charge transferred between the solution and the surface of the glassy carbon electrode continues to increase, as shown in Table 4.

**Table 4:** Scan rate effects on VO^2+^ adsorption and charge quantity (3.13 × 10^− 3^ mol L^− 1^).

Scan rate (V.s^− 1^)	Γ_c_ x10^− 8^ (mol.cm^− 2^)	(-) Q_c_ x10^− 5^ (columb)	Γ_a_ x10^− 8^ (mol.cm^− 2^)	(+) Q_a_ x10^− 5^ (columb)
0.05	0.44	2.69	0.96	5.80
0.02	1.25	7.57	1.41	8.55
0.01	1.86	11.3	2.00	12.1

#### CV of complexation interaction between H_2_TIS ligand and VO^2+^ ions

**Fig. 8:**
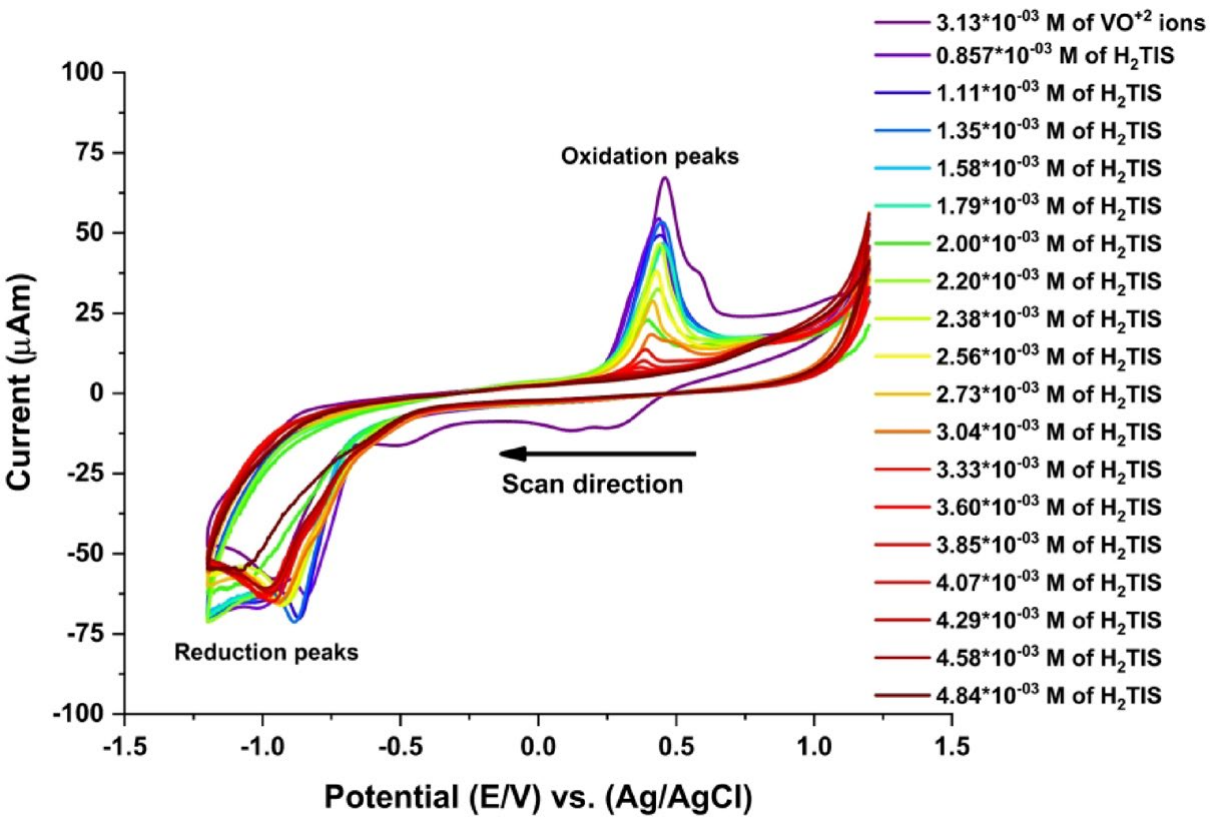
Effect of different H_2_TIS ligand additions on VO^2+^ ion electrochemical behavior in 0.1 M KCl at 303.15 K.

The electrochemical behavior associated with the complexation of VO^2+^ ions by the H_2_TIS ligand was investigated under the experimental conditions shown in Fig. 8. The cyclic voltammograms exhibit systematic shifts of both cathodic and anodic peak potentials toward new values upon increasing ligand concentration, indicating the occurrence of an association reaction between VO^2+^ species and the H_2_TIS ligand. The cyclic voltametric responses obtained for different ligand concentrations were analyzed to assess the effect of complex formation, and the corresponding electrochemical parameters are summarized in Table 5. The stability constants (βj), which quantify the strength of interaction between the ligand and the metal ion, were evaluated using Lingane’s equation (Eq. 8)^42^

8 $$\Delta {\mathrm{E}}^{\circ}{\text{ }}={\text{ E}}^{\circ}{\text{C }} - {\text{ E}}^{\circ}{\text{M }}={\text{ }}\left( {2.303{\mathrm{RT}}/{\mathrm{nF}}} \right){\text{ x }}\left( {{\text{log }}\beta {\text{j }}+j{\text{log }}\left[ {\mathrm{L}} \right]} \right)$$

In this expression, E˚M and E˚C represent the formal potentials of the free metal ion and the metal ligand complex, respectively. R is the gas constant (8.314 J.mol^− 1^.K^− 1^), T is the absolute temperature, n is the number of electrons transferred, F is the Faraday constant (96,485 C.mol^− 1^), [L] is the concentration of the H_2_TIS ligand, and j denotes the stoichiometric coefficient of the complex. The formal potential was calculated according to the conventional midpoint definition (Eq. 9)^43^

9 $${\mathrm{E}}^\circ {\text{ }}={\text{ }}\left( {{\text{Ec }}+{\text{ Ea}}} \right)/2$$

It should be emphasized that, because the voltametric response in Fig. 8 exhibits pronounced quasi-irreversible characteristics (large peak to peak separation and non-unity Ipa/Ipc ratios), the midpoint potential defined by Eq. (8) does not represent a strict thermodynamic formal potential. Instead, it is employed here as an empirical parameter to monitor relative potential shifts associated with metal ligand complex formation. Such an approach has been widely adopted in metal ligand systems exhibiting irreversible or quasi-reversible electrochemical behavior, where midpoint potentials provide a practical basis for comparative analysis of complexation equilibria rather than absolute thermodynamic quantities^30^. Accordingly, the ΔE° values derived in this study reflect relative changes in electrochemical response upon ligand coordination and allow estimation of stability constants within the limitations imposed by irreversible electron transfer kinetics.

**Table 5:** Cyclic voltammetry data for increasing H_2_TIS ligand additions to VO^2+^ ions in 0.05 M KCl.

M x10^−3^ VOSO_4_	M x10^−3^ H_2_TIS	E_*p*.a._ (V)	E_pc_ (V)	ΔE_*p*_ (V)	i_*p*.a._ µA	(-)i_pc_ µA	E° (V)	ΔE° (V)	J
3.13	--	0.457	−0.958	1.415	54.6	25.3	−0.251	--	0
2.86	0.857	0.437	−0.841	1.278	44.4	53.8	−0.202	−0.049	0.3
2.78	1.11	0.443	−0.867	1.310	39.7	62.0	−0.212	−0.039	0.4
2.70	1.35	0.447	−0.883	1.330	43.4	63.9	−0.218	−0.033	0.5
2.63	1.58	0.451	−0.928	1.379	37.3	57.1	−0.239	−0.012	0.6
2.56	1.79	0.453	−0.921	1.374	35.9	58.5	−0.234	−0.017	0.7
2.50	2.00	0.397	−1.057	1.454	14.2	49.8	−0.330	0.080	0.8
2.44	2.20	0.433	−0.925	1.358	22.6	58.3	−0.246	−0.005	0.9
2.38	2.38	0.440	−0.909	1.349	37.6	57.0	−0.235	−0.016	1
2.33	2.56	0.427	−0.930	1.357	29.3	58.7	−0.252	0.001	1.1
2.27	2.73	0.416	−0.948	1.364	20.3	58.6	−0.266	0.016	1.2
2.17	3.04	0.408	−0.937	1.345	10.4	58.0	−0.265	0.014	1.4
2.08	3.33	0.389	−0.994	1.383	6.42	56.2	−0.303	0.052	1.6
2.00	3.60	0.382	−0.999	1.381	3.65	55.4	−0.309	0.058	1.8
1.92	3.85	0.369	−1.005	1.374	1.79	55.7	−0.318	0.068	2
1.85	4.07	0.307	−0.965	1.272	0.91	59.1	−0.329	0.079	2.2
1.79	4.29	0.252	−0.964	1.216	0.60	56.0	−0.356	0.106	2.4
1.69	4.58	0.196	−0.980	1.176	0.52	55.3	−0.392	0.142	2.7
1.61	4.84	0.196	−1.076	1.272	0.00	49.2	−0.440	0.190	3

**Fig. 9:**
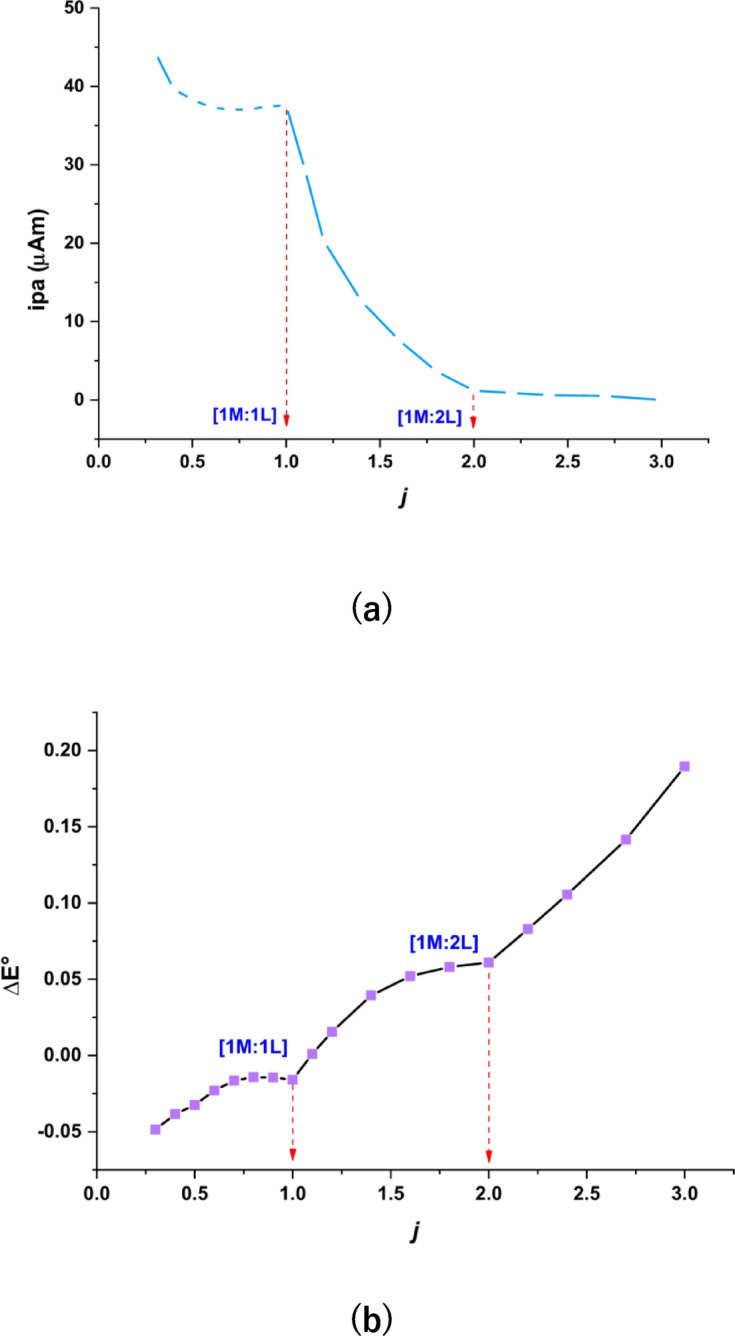
**(a)** Anodic current plotted against *j.* **(b)** ΔE° plotted against *j*.

Using the data from (Epa, Epc, ipa, ipc, E°, j) presented in Table 5, we can evaluate the stoichiometric values of the VO^2+^ complex formed by the addition of the H_2_TIS ligand to the VO^2+^ ion solution through two methods: (i) plotting the anodic peak current (ipa) against the j values in the (V^2+^ to VO^2+^) peak^44^, which reveals breaks at j = 1 and j = 2, indicating two stoichiometric ratios (M: L = 1:1 and 1:2), as shown in Fig. 9a; and (ii) plotting ΔE° against j^45^, illustrated in Fig. 9b, which also presents breaks at j = 1 and j = 2, suggesting the same two stoichiometry for the VO^2+^ complex (M: L = 1:1 and 1:2). Additionally, the stability constants for the 1:1 and 1:2 forms of the VO^2+^ complexes were determined, as depicted in Fig. 9a and b. The Gibbs free energies were calculated using “Lingane’s equation.” (10)^46,47^

10 $$\Delta {\text{G }}={\text{ }} - {\text{ }}2.303{\text{ RT log}}{\beta _j}$$

**Scheme 4:**
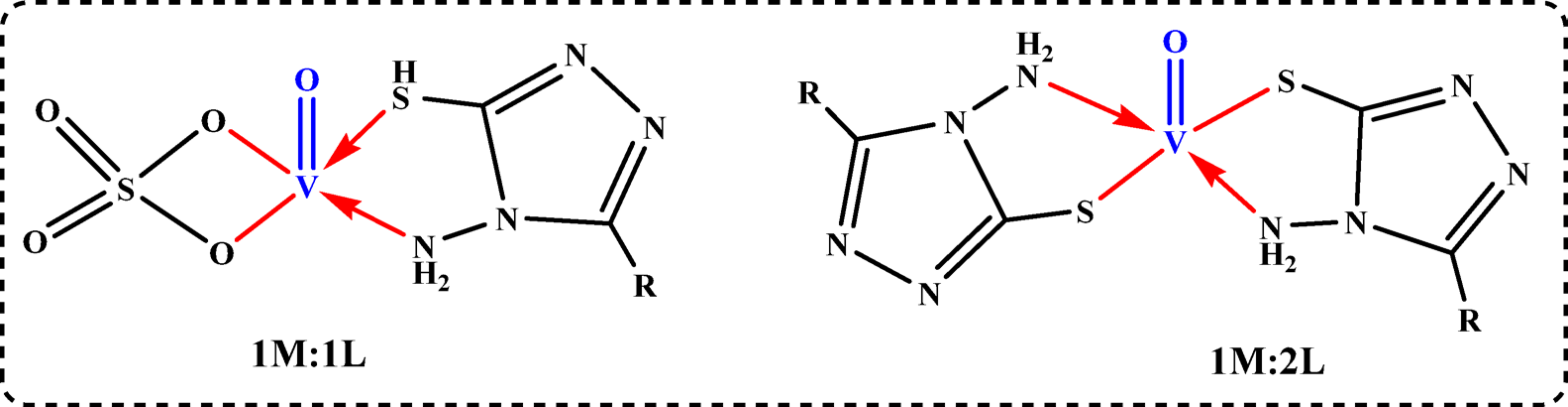
The proposed coordination mode of VO^+ 2^ with H_2_TIS in 0.1 M KCl.

The Gibbs free energies and stability constants in Table 6 indicate that the H_2_TIS ligand forms stable complexes with VO^2+^ in both 1:1 and 1:2 ratios and that these interactions occur spontaneously (Scheme 4).

**Table 6:** Results of the Gibbs free energy and stability constants in the different forms of the VO^2+^-H₂TIS complex.

M x10^− 3^ VOSO_4_	M x10^− 3^ H_2_TIS	j	M: L	log β_j_	β_j_	ΔG kJ.mol^− 1^
2.38	2.38	1.0	1:1	2.100	125.9998	−12.390
1.92	3.85	2.0	1:2	7.038	10,904,107	−41.523

#### Effect of Complexation interaction between VO^2+^ and H_2_TIS on D, n_a_, and k_h_

**Fig. 10:**
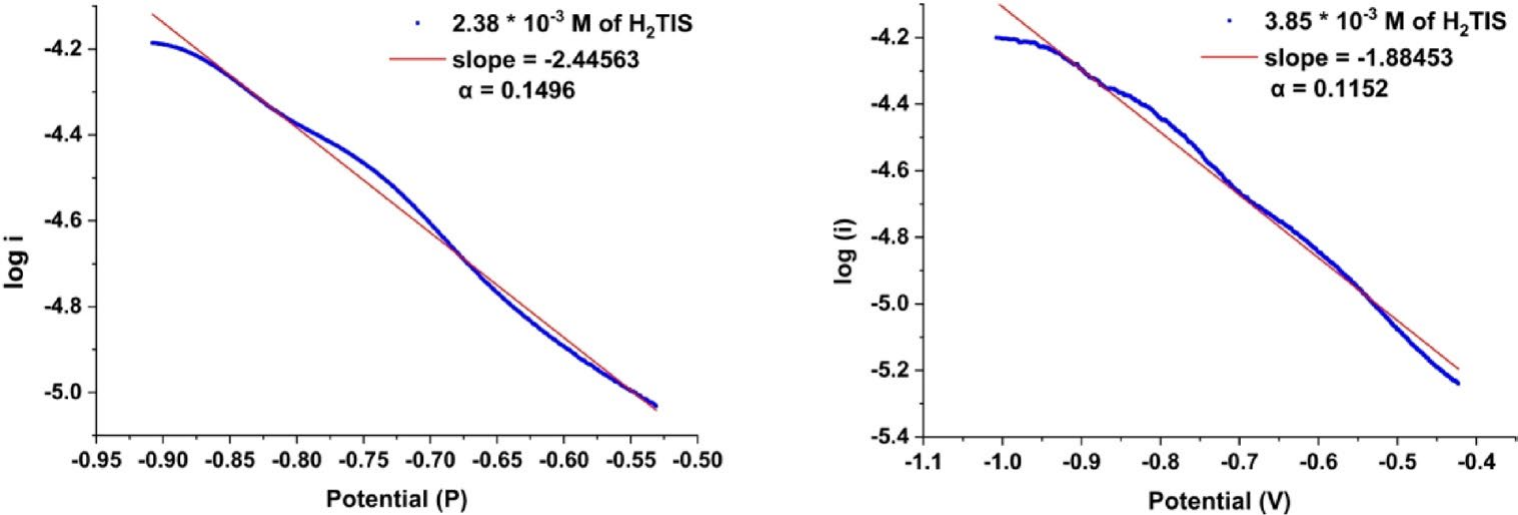
Tafel plot for the complexes forms (M: L, 1:1 and 1:2) of VO^2+^ and (H_2_TIS) ligand.

Cyclic voltammetry was utilized to investigate the complexation behavior of VO^2+^ with H_2_TIS, as illustrated in Fig. 8. The electrochemical parameters obtained from these voltammograms including Epc, Epa, ∆Ep, ipc, ipa, ipa/ipc, Dc, Da, Epc-Epc/2, Epc/2, n_a_, α, and k_h_ are listed in Table 7. The redox process in the complexes formed by the interaction between VO^2+^ and the H_2_TIS ligand exhibits lower irreversibility compared to that of free VO^2+^ ions, as shown in Fig. 10, with charge transfer coefficients of 0.1496 and 0.1152 for the 1:1 and 1:2 ratios, respectively.

Complexation significantly shifts VO^2+^ reduction potentials (Epc) to more negative values (Table 7) and decreases ∆Ep compared to free VO^2+^ ions. Charge transfer coefficients below 0.3 suggest reduced irreversibility, and the standard heterogeneous rate constant (k_h_) for the electron transfer reaction is low (0.01–0.02 cm/s). The rate determining step involves more electrons (n_a_) than in the free VO^2+^ ion case, confirming VO^2+^ ion complexation with H_2_TIS. These results are consistent with the stability constants and Gibbs free energy values, indicating a spontaneous complexation process.

**Table 7:** Cyclic voltammetry results for different forms of the VO^2+^-H_2_TIS complex.

M: L	E_*p*.a._	E_pc_	∆E_*p*_	i_*p*.a._	-i_pc_	i_*p*.a._/i_pc_	D_a_	D_c_	E_pc/2_	E_pc_-E_pc/2_	α	*n*_a_	k_h_ cm.sec^− 1^
	V			µA			cm^2^/sec x10^− 6^		V				
1:1	0.440	−0.909	1.349	37.6	57.0	0.659	9.039	20.79	−0.767	1.676	0.150	0.197	0.023
1:2	0.369	−1.005	1.374	1.79	55.7	0.032	0.032	30.50	−0.793	1.798	0.115	0.238	0.011

## Effect of complexation interaction between VO^2+^ and H_2_TIS on Γ and Q

The complexation formation between the H_2_TIS ligand and the VO^2+^ ions and results in an inhibition in adsorption and the quantity off charge compared to the event of free VO^2+^, as seen in Table 8.

**Table 8:** Complexation effects on adsorption and charge quantity.

M: L	Γ_c_ x10^− 8^ (mol. cm^− 2^)	(-) Q_c_ x10^− 5^ (columb)	Γ_a_ x10^− 9^ (mol. cm^− 2^)	(+) Q_a_ x10^− 5^ (columb)
1:1	0.99	6.05	6.59	3.99
1:2	0.97	5.92	0.31	0.19

### Spectrophotometric studies

#### Job’s method (continuous variation)

**Fig. 11:**
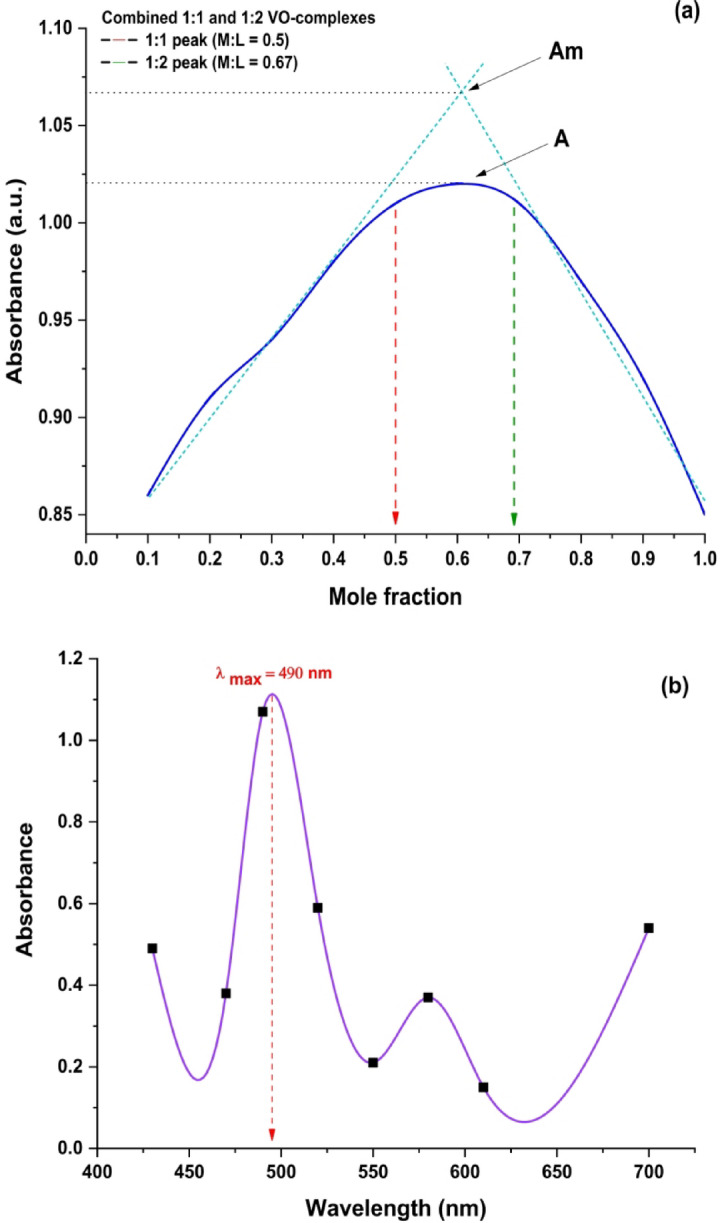
**(a)** Job’s plot and, (**b)** Maximum wavelength (λ_max_) for formed 1:1, 1:2 of VO^2+^-complexes.

Job’s technique of continuous variation was applied to determine the stoichiometry of the metal complexes^48^. This approach relies on measuring the absorption of a series of solutions where the molar concentrations of two reactants fluctuate, but their total stays constant. Job’s method is alternatively referred to as the method of continuous variation. The method’s premise involves varying the mole ratio of the metal ion to the ligand between 0 and 1 while maintaining a constant total concentration, C = C_metal_ + C_ligand_. The absorbance of each combination was measured after the equilibration of the reaction mixtures (M & L). The absorbance of each solution was graphed versus the ligand mole fraction ([L]/[M]+[L])^49^. By plotting the graph of absorbance against the corresponding mole fraction of the produced series of solutions, the precise ratio of metal to ligand at equilibrium may be seen at the peak of the curve. The peak absorbance, shown by the curve of continuous variation, was seen at ligand mole fractions ([L]/[M]+[L]) of 0.5 and ≈ 0.67, implying complex formation at 1:1 and 1:2 (M: L) molar ratios, respectively, as shown in Fig. 11a and Table 9b. Additionally, the maximum wavelength of the complex was determined by plotting absorbance against wavelength. The absorption wavelengths were established at 490 nm, as seen in Fig. 11b and Table 9a.

**Table 9:** Data of (**a**) maximum wavelength (λ_max_) and (**b**) Job’s continuous variation method of VO^2+^-H_2_TIS complex.

a		B		
λ (nm)	Absorbance at [1 M:1 L]	M: L [1 × 10^− 3^ M]	M/L= V_L_/(V_L_+V_M_)	Absorbance at λmax = 490 nm
430	0.49	(9:1)	0.1	0.86
470	0.38	(8:2)	0.2	0.91
490	1.02	(7:3)	0.3	0.94
520	0.59	(6:4)	0.4	0.98
550	0.21	(5:5)	0.5	1.01
580	0.37	(4:6)	0.6	1.02
610	0.15	(3:7)	0.7	1.01
700	0.54	(2:8)	0.8	0.97
--	--	(1:9)	0.9	0.92
--	--	(0:10)	1	0.85

#### Stability constant

Evaluation of the stability constants for the VO^2+^-complexes formed from the interactions between the H_2_TIS ligand and VO^2+^ ions in solution was a significant objective of our study to elucidate the coordination behavior towards the chosen VO^2+^ ions. The spectrophotometric measurements obtained via Job’s continuous variation approach were used to ascertain the formation constants of the metal complexes generated in solution^50^, as shown in Fig. 11a. The findings indicate that a straightforward equilibrium model for the production of metal complexes between the H_2_TIS ligand and the chosen metal ion may be expressed as VO^2+^ + H_2_TIS ⇌ [VO^2+^-H_2_TIS] for 1:1 and 1:2 stoichiometry. Furthermore, the complex formation constant (K_f_) may be assessed using the theoretical relation (11)^51,52^ provided as:

11 $${{\mathrm{K}}_{\mathrm{f}}}={\text{ }}\left( {{\text{A }}/{\text{ Am}}} \right){\text{ }}/{\text{ }}{{\mathrm{C}}^{\mathrm{n}}}{{\mathrm{n}}^{\mathrm{n}}}{\left[ {1 - {\text{ }}\left( {{\text{A }}/{\text{ Am}}} \right)} \right]^{{\mathrm{n}}+1}}$$

Where A represents the found maximum absorbance (1.02), Am refers to the absorbance determined from the extrapolation of the two lines acquired from Job’s continuous variation curve (1.07), C reveals an initial molar concentration of the studied metal ion, and n refers to the stoichiometric ratio of the complex^53^. Furthermore, the formula ΔG = -RTlnK allows for the estimation of the Gibbs free energy (ΔG, kJ mol^− 1^) associated with the production of metal complexes, where R represents the gas constant (8.314 J.mol^− 1^.K^− 1^), T denotes the temperature in Kelvin, and K signifies the determined stability constant^54^. The determined values of K_f_ for the interactions of H_2_TIS with VO^2+^ ions were 4.22 × 10^3^ and 4.13 × 10^4^ for 1:1 and 1:2 stoichiometry, respectively. Furthermore, the derived negative values of ΔG for the same system are − 2.10 × 10^4^ and − 2.51 × 10^4^ for the 1:1 and 1:2 ratios, respectively. The negative values of ΔG indicate that the kinetic process is spontaneous^55^.

#### Molar ratio method

Spectrophotometry, employing the molar ratio method (constant metal ion concentration, varied ligand concentration), determined the molecular structures and stability constants of colored complexes. Absorbance measurements at a fixed wavelength were plotted against the ligand to metal ion molar ratio. Intersections of resulting linear segments revealed the molar ratio of the most stable complexes. Absorbance measurements were taken at a fixed wavelength, and the resulting data was graphed as absorbance versus ligand-to-metal ion molar ratio. Intersections of the linear segments indicate the molar ratios of the most stable complexes^56^.

**Fig. 12:**
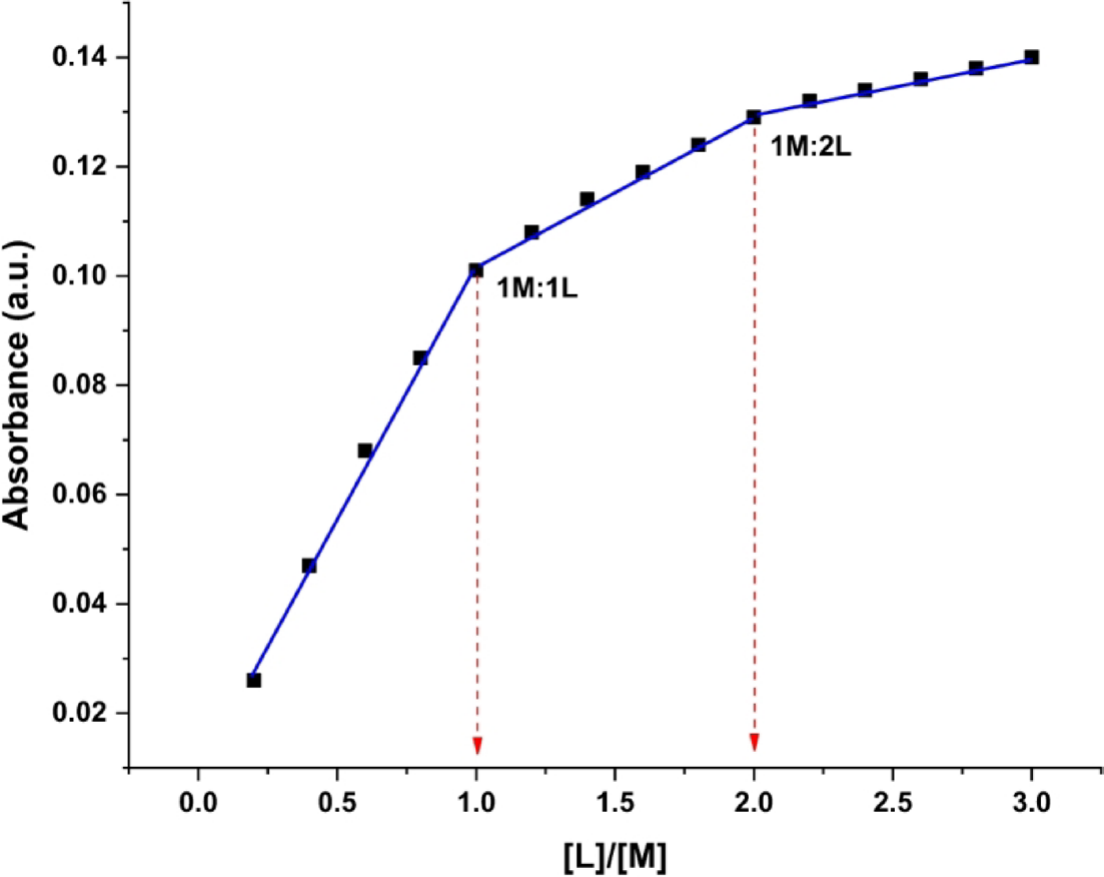
Mole ratio plot for formed 1:1, 1:2 of VO^2+^-complexes.

The mole ratio approach was used to ascertain the stoichiometry of the complexes (VO^2+^: H_2_TIS ratio) present in solution. The stability constants of the complexes in solution were determined using a mole ratio approach. Table 10 presents the various concentrations of ligand (1.0 × 10^− 4^ M) combined with a constant VO^2+^ concentration (5.0 × 10^− 4^ M) at 490 nm. Figure 12 illustrates a distinct break at a 1:1 and 1:2 mol ratios of VO^2+^-H_2_TIS complexes in solution.

**Table 10:** The Absorbance of VO^2+^-H_2_TIS system at λ_max_ = 490 nm using the mole ratio method.

[L]/[M]	0.2	0.4	0.6	0.8	1.0	1.2	1.4	1.6	1.8	2.0	2.2	2.4	2.6	2.8	3.0
**Abs. of ** **VO** ^**2+**^ **–H**_**2**_ **TIS**	0.026	0.047	0.068	0.085	0.101	0.108	0.114	0.119	0.124	0.129	0.132	0.134	0.136	0.138	0.140

## Conclusions

The electrochemical investigation demonstrates that the redox behavior of the H_2_TIS ligand is strongly medium dependent and governed by the redox activity of its functional groups. In acidic medium (HNO_3_), the cathodic process is attributed to reduction of the indolin-2-one carbonyl group (C = O) to its hydroxyl form (C-OH), while the anodic response originates from ligand-centered oxidation of the thiol group (-SH) to the thione form (C = S) *via* a proton-coupled electron transfer process. In neutral medium (KCl), the carbonyl moiety exhibits quasi-reversible redox behavior, indicating electrochemical stability under near-neutral conditions. In alkaline medium (NaOH), the thiol group is predominantly deprotonated to a thiolate species (L-S^−^), leading to an irreversible anodic process assigned to thiolate oxidation and subsequent coupling of sulfur centered intermediates to form disulfide-linked dimeric species, with no significant corresponding reduction peak. The electrochemical behavior of free VO^2+^ ions in KCl solution exhibits a quasi-reversible VO^2+^ centered redox response; however, coordination with H_2_TIS induces distinct changes in the voltammetric profiles and enhances the electrochemical stability of the metal center. Combined cyclic voltammetry, Job’s method, and molar ratio analyses suggest the formation of both 1:1 and 1:2 VO^2+^-H_2_TIS complexes in solution, with the calculated stability constants and negative Gibbs free energy values indicating spontaneous complex formation with partial covalent character. Overall, these results highlight the medium dependent redox versatility of H_2_TIS and its strong coordination ability toward VO^2+^ ions, underscoring its potential applicability in electrochemical sensing platforms.

## Data Availability

The data supporting the research findings can be obtained from the corresponding author, upon reasonable request.
